# Outcomes of dual mobility cemented versus cementless cups in primary total hip arthroplasty: a systematic review and meta-analysis

**DOI:** 10.1530/EOR-2025-0039

**Published:** 2026-06-01

**Authors:** Michael Silveira Santiago, Fatemeh Akbarpoor, Felipe J Aidar, Obada Tarabichi, Carlos Lopez-Villalobos, Reuthemann Esequias Teixeira Tenorio Albuquerque Madruga

**Affiliations:** ^1^ Health Sciences Graduate Program, Federal University of Sergipe, Aracaju, Brazil; ^2^ College of Medicine, Mohammed Bin Rashid Univertsity of Medicine and Health Sciences, Dubai, UAE; ^3^ Federal University of Sergipe, São Cristóvão, Brazil; ^4^ Department of Physical Education, Federal University of Sergipe, São Cristóvão, Brazil; ^5^ University of Sharjah, Sharjah, UAE; ^6^ University of Costa Rica, San Jose, Costa Rica; ^7^ Department of Medicine, Federal University of Sergipe, Aracaju, Brazil

**Keywords:** acetabular, cemented, cementless, cups, dual mobility

## Abstract

**Purpose:**

- With the increased use of dual mobility primary hip arthroplasty with cementless and cemented cups, we found different clinical and radiographic responses. The goal of this systematic review is to compare the types of acetabular fixation according to clinical and radiographic changes.

**Methods:**

- We conducted a pairwise analysis comparing cementless versus cemented cups in a dual mobility hip arthroplasty. The outcomes of interest were pain at rest, pain during activity, functional score, wear rate, and cup migration up to 7 years of follow-up. Of the 596 studies identified, only 3 were eligible for inclusion in the study. Our search strategy was broad and carefully designed to capture all relevant studies.

**Results:**

- We included two randomized controlled trials and one cohort study, amounting to 233 patients, of which 108 used cemented cups and 125 received cementless cups. There was no statistical significance (*P* > 0.05) in all outcomes analyzed between both types of acetabular fixation: pain at rest (SMD: 0.17; 95% CI: – 0.21 to 0.56), pain during activity (SMD: 0.36; 95% CI: – 0.03 to 0.74), functional score (MD: 1.72; 95% CI: – 0.51 to 3.95), cup migration (SMD: – 0.45; 95% CI:−0.96 to 0.05), and wear rate (MD: −0.13; 95% CI: −0.40 to 0.13).

**Conclusion:**

- Dual mobility primary total hip arthroplasty, whether using a cemented or uncemented cup, represents an effective treatment option, yielding similar clinical and radiographic outcomes.

## Introduction

Total hip arthroplasty (THA) is a commonly performed procedure in the field of orthopedic surgery, which is cost-effective and successful in most patients (1). Despite its widespread success, THA is not without complications, which can significantly impact patient outcomes and implant longevity. Common complications include dislocation, prosthetic joint infection, periprosthetic fracture, heterotopic ossification, and component loosening or wear (2, 3). Of these, dislocation remains a key concern, particularly in high-risk populations, including patients with neuromuscular disorders, those with recurrent instability, or those undergoing revision surgeries (4, 5).

The type of acetabular cup used in THA plays a crucial role in mitigating complications, especially dislocation. Traditional fixed-bearing cups (non-dual mobility) provide reliable outcomes but may pose a higher risk of dislocation in specific patient groups. Dual mobility (DM) cups were introduced to address this issue by incorporating an additional polyethylene articulation, which increases the jump distance and reduces impingement, thus lowering the risk of dislocation (6, 7). Several studies have reported the superiority of DM cups (DMCs) in reducing instability, particularly in revision THA and high-risk primary THA patients (8, 9). The use of dual mobility in primary hip arthroplasty has increased over the years due to the need to increase the stability of the prosthetic set and consequently reduce the risk of dislocations. Initially, this type of arthroplasty was primarily favored for elderly patients with neurological conditions, proximal femur fractures, and/or muscular deficiencies. However, its indications have expanded over time to reduce complications, driven by advancements in prosthetic design, acetabular fixation, and material improvements (10, 11). Depending on the bone porosity of the host bone and the surgeon’s experience, cemented or cementless fixation of the acetabular cup can be used in dual mobility. Recent studies have shown lower complication rates of this type of arthroplasty compared to the conventional forms of prosthetic implants (12). With technological advancements, highly cross-linked polyethylene was introduced for the mobile component, preventing increased wear, osteolysis, or aseptic loosening in modern cementless dual mobility cups (DMCs). In elderly patients with poor acetabular bone quality, cemented acetabular fixation is often preferred to enhance primary stability while minimizing additional reaming of the acetabular diameter (13, 14).

With the growing use of dual mobility arthroplasty with both cementless and cemented cups, varying clinical and radiographic outcomes have been observed. This systematic review aims to compare these two types of acetabular fixation in terms of clinical and radiographic changes, as well as functional outcomes.

## Materials and methods

This systematic review and meta-analysis was registered in the International Prospective Register of Systematic Reviews and Clinical Trials (PROSPERO) under protocol number CRD42025644784 to promote transparency and reduce the risk of bias. The study adhered to the guidelines of the Cochrane Collaboration Handbook for Systematic Reviews of Interventions and the Preferred Reporting Items for Systematic Reviews and Meta-Analyses (PRISMA) statement (15, 16, 17).

### Eligibility criteria

In the study selection process, we started with deduplication and independently screened all potentially eligible studies. Two independent reviewers and one validator collaborated in combining outcomes from three databases and evaluated the studies for inclusion. We excluded articles based on title and abstract if they did not pertain to the subject of interest. The remaining studies were then reviewed in full to verify eligibility. We excluded trials not concluded, narrative reviews, systematic reviews, non-comparative research, scientific posters, study protocols, conference abstracts not peer-reviewed, any pre-clinical studies, or those not published in English.

We finished with a full independent reading of the papers by two authors with the following inclusion criteria: (i) peer-reviewed, (ii) compared DM cemented versus DM cementless, and (iii) involved primary hip arthroplasty. Studies were excluded if they (i) were ongoing trials, not concluded, (ii) were basic science research, and (iii) did not provide data on each outcome chosen. We made no exclusions related to the publication date. Data extraction was manually performed.

Discrepancies were resolved through consensus between the reviewers, and a third author made the final decision in the event of divergence.

### Search strategy and data extraction

We systematically searched PubMed, Embase, and Cochrane Central Register of Controlled Trials databases from inception to January 2025. We included a combination of Medical Subject Headings terms and keywords relating to ‘dual mobility’, acetabular, cemented, cementless, uncemented, and cups (Supplementary Table 1 (see section on Supplementary materials given at the end of the article)). The references from all included studies, previous systematic reviews, and meta-analyses were also searched manually for any additional studies. Two authors independently extracted the data following predefined search criteria and quality assessment.

### Endpoints (outcomes)

For continuous outcomes, the pooled analysis was conducted to compare mean differences (MDs) or standardized mean differences (SMDs) with 95% confidence intervals (CIs) between cemented and cementless dual mobility cups. A random-effects model was used to account for potential heterogeneity across studies. Heterogeneity was assessed using the *I*^2^ values of 25, 50, and 75%, representing low, moderate, and high heterogeneity, respectively (18).

The outcomes of interest included wear rate, cup migration, functional scores, and pain (at rest and during activity). The pooled MD was calculated for outcomes reported on the same scale, while SMD was used for outcomes reported on different scales. Due to variability in the scales used across studies to measure outcomes such as pain and function (VAS, HHS, and WOMAC), we used SMD in our meta-analysis. The SMD enables comparison of effect sizes across studies that report the same outcome using different measurement tools by expressing the intervention effect in units of standard deviation. This approach allows for synthesis of heterogeneous outcome measures while maintaining comparability. A *P*-value of <0.05 was considered statistically significant. Statistical analyses were performed using R Studio 4.4.0.

### Quality assessment

Two review authors assessed the risk of bias in each study using the revised Cochrane risk-of-bias (RoB 2/ROBINS-I) tools. The examination domains included biases arising from the randomization process, deviations from intended interventions, missing outcome data, measurement of the outcome, and selection of the reported result. After responding to the signaling questions, one of three types of bias judgments was selected, namely ‘low’, ‘high’, and ‘some concerns’. In case of conflicts, a third author was contacted as an unbiased arbitrator (19). The layout was generated using RobVis (20). The overall quality of evidence was evaluated following the Grading of Recommendations, Assessment, Development and Evaluation (GRADE) guidelines (21).

### Statistical analysis

Relative risk (RR) with the Mantel–Haenszel method was used to calculate binary outcomes. MDs were used when scales were the same; otherwise, a SMD was applied, both using the inverse method as explained in the section titled ‘Endpoints (outcomes)’. The restricted maximum likelihood random effect was applied to synthesize the pooled analysis along with 95% CIs. Cochran *Q* test and *I*^2^ statistics were used to assess heterogeneity; *P* values inferior to 0.10 and *I*^2^ > 25% were considered significant for heterogeneity. R (version 4.4.0) was used for all statistical analyses.

## Results

### Study selection and characteristics

**Figure 1:**
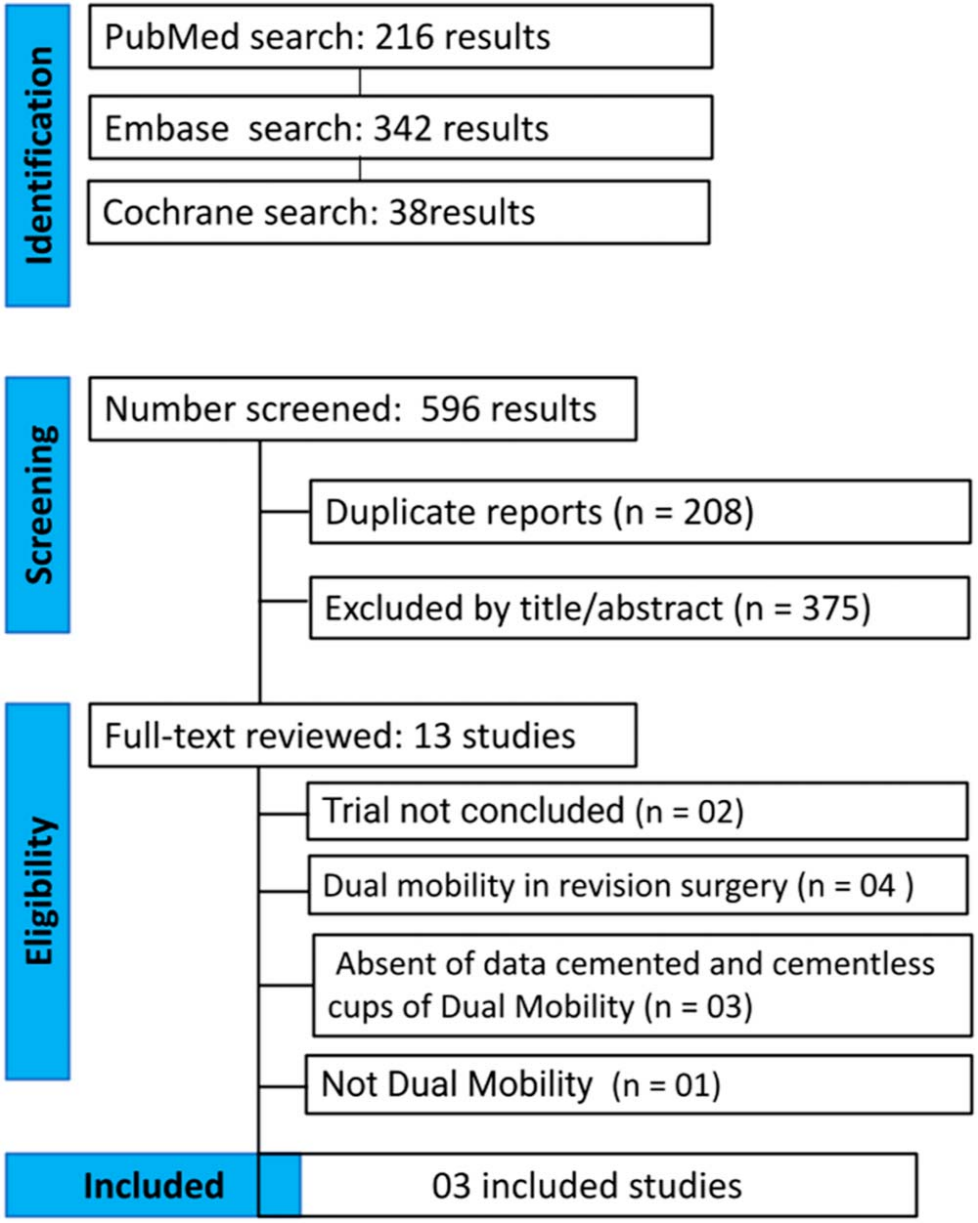
PRISMA flow diagram of study screening and selection.

As detailed in Fig. 1, the initial search identified 596 studies, of which 208 duplicates and 375 ineligible studies were excluded. Following a full-text review of the remaining 13 articles, 10 studies were excluded based on the predefined criteria (Fig. 1). Ultimately, three studies comparing dual mobility and conventional THA for femoral neck fractures were included in the analysis (22, 23, 24). A total of 233 patients were included, of which 108 (46.35%) patients received cemented DM and 125 (53.65%) received cementless dual mobility cups. The mean follow-up time ranged from 2 to 7 years, and the mean age of included patients ranged from 74 to 78 years. The baseline characteristics are summarized in Table 1. Of the three studies included in our systematic review, two were randomized clinical trials and one was a retrospective cohort study.

**Table 1:** Baseline characteristics of the included studies.

Study/group	Study design	Cause	Sample size	Female	Mean FU (years)	Mean age (years)	Mean BMI
Jorgensen *et al.* (22)	RCT	OA			6	74	28
Cemented			23	13			
Cementless			22	11			
Tabori-Jensen *et al.* (24)	Cohort	FNF					MD
Cemented			56	46	7.6	78	
Cementless			73	51	7.7	75	
Tabori-Jensen *et al.* (23)	RCT	OA			2	75	
Cemented			29	15			28
Cementless			30	17			29

RCT, randomized controlled trial; OA, osteoarthritis; FNF, femoral neck fracture; MD, missing data; and FU, follow-up.

### Pooled analysis of all studies

**Figure 2:**
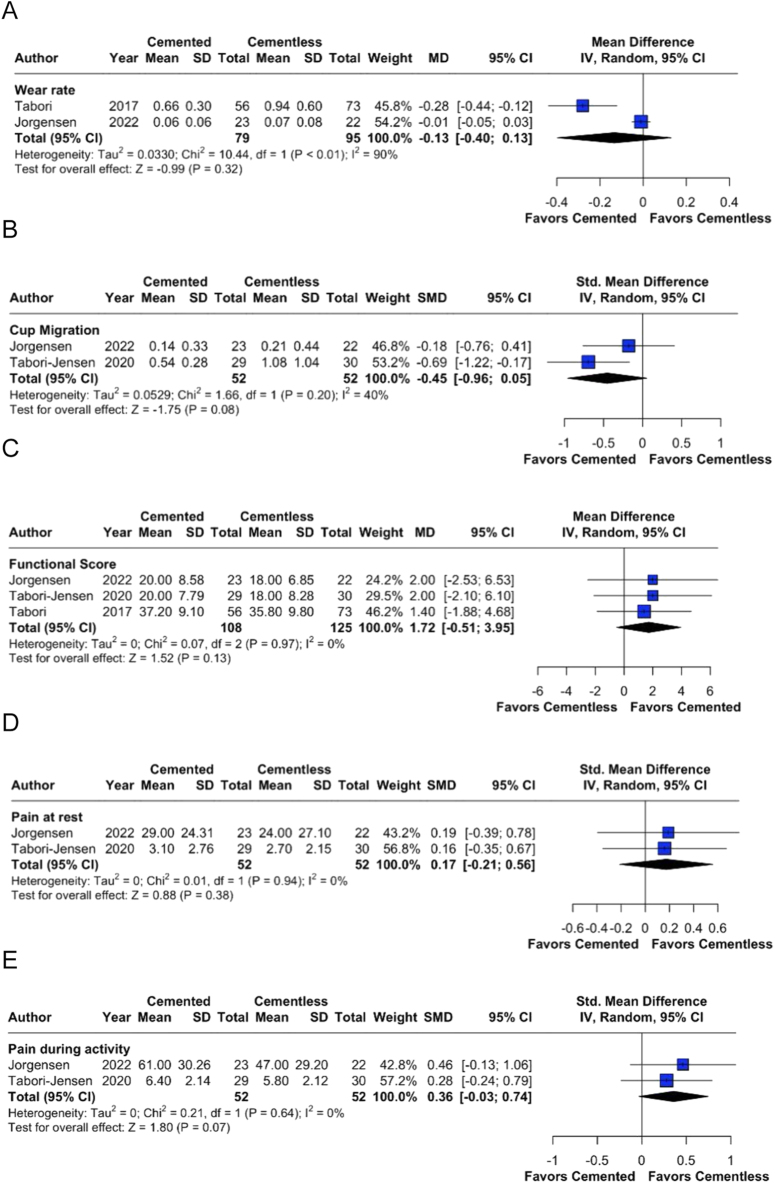
Forest plots for the following endpoints: (A) wear rate, (B) cup migration, (C) functional score, (D) pain at rest, and (E) pain during activity.

Two studies reported wear rate, with results indicating no statistically significant difference between cemented and cementless cups (MD: −0.13; 95% CI: −0.40 to 0.13; *P* = 0.32). The heterogeneity was high (*I*^2^ = 90%), likely due to the differences in follow-up durations and measurement techniques (Fig. 2A). The analysis of cup migration included two studies, suggesting a nonsignificant trend toward reduced migration in cemented cups (SMD: – 0.45; 95% CI:−0.96 to 0.05; *P* = 0.08). Moderate heterogeneity was observed (*I*^2^ = 40%), reflecting variability in study populations and outcome definitions (Fig. 2B).

Three studies reported functional scores, indicating no significant difference in functional outcomes between cemented and cementless fixation (MD: 1.72; 95% CI: – 0.51 to 3.95; *P* = 0.13). No heterogeneity was observed (*I*^2^ = 0%), suggesting consistency in findings across studies (Fig. 2C). Two studies assessed pain at rest, and we found no statistically significant difference between cemented and cementless fixation (SMD: 0.17; 95% CI: – 0.21 to 0.56; *P* = 0.38). There was no heterogeneity (*I*^2^ = 0%) (Fig. 2D).

Two studies evaluated pain during activity, and the results suggest a trend favoring cementless fixation with less pain, although this result did not reach statistical significance (SMD: 0.36; 95% CI: – 0.03 to 0.74; *P* = 0.07). No heterogeneity was observed (*I*^2^ = 0%) (Fig. 2E).

### Quality assessment and publication bias

The risk-of-bias assessment for the study by Tabori-Jensen (2017) was conducted using the ROBINS-I tool, evaluating seven domains (24). Domain 1 (bias due to confounding) was judged as moderate risk, represented by a yellow circle, indicating that some confounders might not have been fully addressed. All other domains, including bias due to the selection of participants (D2), bias in the classification of interventions (D3), bias due to deviations from intended interventions (D4), bias due to missing data (D5), bias in the measurement of outcomes (D6), and bias in the selection of the reported result (D7), were rated as low risk, as denoted by green circles. Overall, the study was assessed as having a moderate risk of bias, suggesting that while most methodological aspects were rigorous, confounding remains a concern that could influence the validity of the study’s findings (Supplementary Fig. 1A).

The risk of bias for the two included RCTs was assessed using the risk-of-bias 2 (RoB 2) tool, which evaluates five domains: bias arising from the randomization process, bias due to deviations from intended interventions, bias due to missing outcome data, bias in the measurement of the outcome, and bias in the selection of the reported result. The studies by Jorgensen (2022) and Tabori-Jensen (2020) were both rated as having a low risk of bias in all domains, as indicated by the green plus symbols in the assessment. Consequently, the overall risk of bias for both studies was judged to be low, indicating a high level of methodological rigor and supporting the validity of their findings (22, 23) (Supplementary Fig. 1B).

In the GRADE and summary of contents table, the comparison between cemented and cementless dual mobility acetabular cups in primary THA demonstrated varying outcomes across multiple domains, with no statistically significant differences observed. Overall, the certainty of evidence was rated as moderate, primarily due to imprecision and heterogeneity (Supplementary Fig. 2). A nonsignificant trend toward lower pain during activity was observed in the cementless group (SMD = 0.36; 95% CI: −0.03 to 0.74; *P* = 0.07), while no differences were observed in pain at rest or functional outcomes. Functional scores were supported by moderate certainty evidence, with a MD of 1.72 points (95% CI: 0.51–3.95), influenced by the inclusion of one observational study. For radiographic outcomes, nonsignificant trends favoring cemented dual mobility cups were observed for cup migration (SMD: −0.45; 95% CI: −0.96 to 0.05) and wear rate (MD: −0.13; 95% CI: −0.40 to 0.13); however, these findings were characterized by wide confidence intervals and substantial heterogeneity. Collectively, these results suggest comparable short- to mid-term clinical and radiographic outcomes between fixation methods and underscore the need for larger, high-quality studies with longer follow-ups to clarify potential differences in fixation performance.

A funnel plot was not generated in this analysis due to the inclusion of fewer than ten studies (25). With fewer studies, the plot may lack interpretive reliability and could lead to misleading conclusions. Consequently, we opted not to perform this analysis to maintain the robustness and validity of the results.

## Discussion

In this systematic review and meta-analysis of two randomized controlled trials and one retrospective cohort study encompassing 233 patients, we compared the outcomes in patients who received cemented and cementless DM cups. Our main findings are that there was no statistically significant difference in wear rate, cup migration, functional scores, or pain outcomes (both at rest and during activity) between the two groups. While the analysis of cup migration showed a trend favoring cemented fixation and pain during activity showed a trend favoring cementless fixation, neither reached statistical significance. Overall, both cemented and cementless dual mobility cups demonstrated comparable clinical and radiographic outcomes.

Wear rate is a critical factor affecting the longevity of THA. Excessive polyethylene wear can lead to implant loosening, osteolysis, and the need for revision surgery. Previous studies have suggested that polyethylene wear in DM cups is primarily influenced by the quality of the implant design rather than the type of fixation (6). Prudhon *et al*. (2013) similarly reported that long-term wear rates remained consistent between cemented and cementless DM cups (26). Our study aligns with these findings, showing no significant difference in wear rates between cemented and cementless DM cups. This suggests that both fixation methods provide a comparable level of protection against wear-related complications, supporting the reliability of DM cups in reducing polyethylene wear regardless of fixation type.

Cup migration can compromise implant stability and lead to the failure of THA. Early migration is particularly concerning, as it may predict long-term loosening. Studies have shown that cemented fixation offers better initial stability, especially in patients with poor bone quality (27). In contrast, cementless fixation has been associated with excellent long-term outcomes in younger, healthier patients due to its potential for osseointegration (9). Nonetheless, unlike the study by Laende *et al.* (2019), we found a stable migration of the acetabular dome in a follow-up of up to 7 years, which brings a low risk of aseptic loosening and low wear rates and a greater security in the viability of the prosthetic implant (28). This current study revealed a nonsignificant trend favoring cemented fixation for reduced early migration, which is consistent with the finding that cemented cups may provide more reliable early stabilization. However, the comparable performance of cementless cups in our study suggests that modern cementless designs have closed the gap in achieving early stability.

Considering functional recovery is a key goal of THA, determine an improvement in mobility and an increase in quality of life. Studies have consistently shown that DM cups result in significant functional improvements, regardless of the fixation methods (29). No differences in functional recovery have been observed between cemented and cementless DM cups in mid-term follow-up (30). The present meta-analysis found similar results, as no significant difference was observed in functional scores between the two groups. This reinforces the notion that both cemented and cementless DM cups can provide substantial improvements in physical function and patient-reported outcomes.

Pain relief is one of the primary objectives of THA, directly influencing patient satisfaction. Effective pain reduction after THA is often attributed to proper implant alignment and biomechanical performance rather than fixation type. Studies have reported that both cemented and cementless DM cups provide comparable pain relief at rest and during activity (9). Similarly, our analysis showed no significant difference in pain outcomes between the two groups. These results suggest that both fixation methods are effective in managing pain, allowing surgeons the flexibility to choose based on other patient-specific factors.

Furthermore, similar to our results, cemented fixation of the acetabular cup has also been a viable alternative in complex cases of osteoarthritis and femoral neck fracture in the elderly (>75 years) and who have a high risk of instability, with high survival rates and a low number of complications (31, 32, 33).

### Clinical implications of findings

The clinical implications of our findings are significant for guiding surgical decision-making in DM during THA. Given the comparable outcomes between cemented and cementless DM cups, the choice of fixation should be based on patient-specific factors, including bone quality and risk profile.

Cemented fixation may be more suitable for patients with poor bone quality, such as those with osteoporosis, to achieve better initial stability. In contrast, cementless fixation may be preferred in younger patients with good bone quality, given its potential for long-term osseointegration and avoidance of cement-related complications. The absence of a significant difference in wear rate and migration suggests that both fixation methods can provide durable and stable constructs. Functional outcomes and pain relief were also comparable, reinforcing the reliability of both techniques in restoring patient mobility and improving the quality of life. Ultimately, these findings provide reassurance that either fixation method can deliver favorable results and the surgical approach can be tailored to individual patient needs and surgeon expertise.

### Limitations and future directions

This meta-analysis has multiple limitations. First, the number of published trials available on databases was a significant impediment in formulating this systematic review, with many of the included studies rated as low confidence and not in accordance with the Consolidated Standards of Reporting Trials (CONSORT) (The CONSORT Statement, 2022), which may limit the reliability of the conclusions. Three studies limit the statistical power and generalizability of our findings. Even considering the limited number of comparative studies, these results should be interpreted as a synthesis of the current evidence rather than as definitive proof of equivalence between cemented and cementless fixation. Additionally, significant heterogeneity was observed in key outcomes, particularly wear rate (*I*^2^ = 90%) and cup migration (*I*^2^ = 40%), reflecting variations in study populations, surgical techniques, follow-up durations, and outcome assessment methods. This variability limits the certainty of pooled estimates and underscores the need for more standardized methodologies in future research. Furthermore, the small number of studies precluded a formal evaluation of publication bias, increasing the risk of undetected small-study effects. Future randomized controlled trials (RCTs) should aim to improve statistical power, ideally with multicenter designs that can recruit a larger, more diverse patient population. A sample size of at least 100 participants per group would be more robust and would provide greater confidence in the findings. Additionally, longer follow-up durations are necessary (e.g. ≥2 years) to evaluate the long-term effects of fixation methods on migration, wear rates, and functional outcomes. Given the heterogeneity observed in the current studies, future RCTs should also focus on stratified or subgroup analyses to evaluate different fixation methods across specific patient groups in order to identify which population would most benefit from cemented vs cementless fixation. In addition, to minimize bias, double-blinded controlled trials with clear inclusion and exclusion criteria would also strengthen the validity of future results.

Finally, differences in patient demographics, surgeon experience, and implant selection criteria may have contributed to residual confounding. Given these limitations, further high-quality randomized trials with larger sample sizes, standardized protocols, and long-term follow-ups are essential to establish definitive conclusions regarding the optimal fixation strategy for dual mobility cups in primary THA.

## Conclusion

In this meta-analysis, available evidence suggests that dual mobility acetabular cups used in primary THA provide comparable short- to mid-term clinical and radiographic outcomes when implanted using either cemented or cementless fixation. No statistically significant differences were observed between fixation methods across the analyzed outcomes. However, the certainty of evidence was predominantly moderate, limited by small sample sizes, heterogeneity, and imprecision. As a result, definitive conclusions regarding the long-term fixation performance of cemented versus cementless dual mobility cups cannot yet be drawn. Further well-designed prospective randomized trials with longer follow-ups are required to better evaluate implant fixation, durability, and long-term clinical outcomes.

## Supplementary materials



## ICMJE Statement of Interest

All authors report no relationships that could be construed as a conflict of interest. All authors take responsibility for all aspects of the reliability and freedom from bias of the data presented and their discussed interpretation.

## Funding Statement

This work did not receive any specific grant from any funding agency in the public, commercial, or not-for-profit sector.
