# Supplementary material for: Outcomes of dual mobility cemented versus cementless cups in primary total hip arthroplasty: a systematic review and meta-analysis
Source: EFORT Open Rev. 2026 Jun 1;11(6):545–52. doi: 10.1530/EOR-2025-0039 (PMC13238971; doi:10.1530/EOR-2025-0039)
Supplement: Supplementary file 1 [file supplementary_materials.pdf]

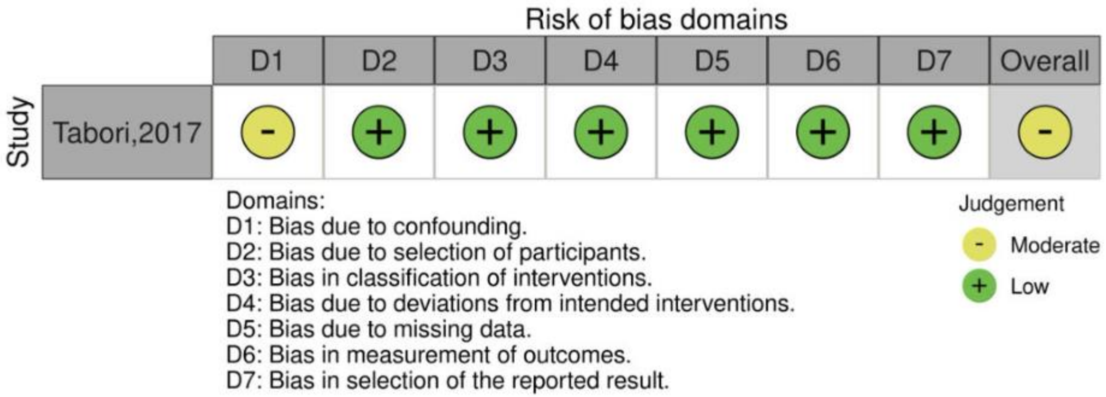

SUPPLEMENTARY FIGURE 1a. Cochrane Risk-of-Bias applied in observational study of interventions (**ROBINS - I**)

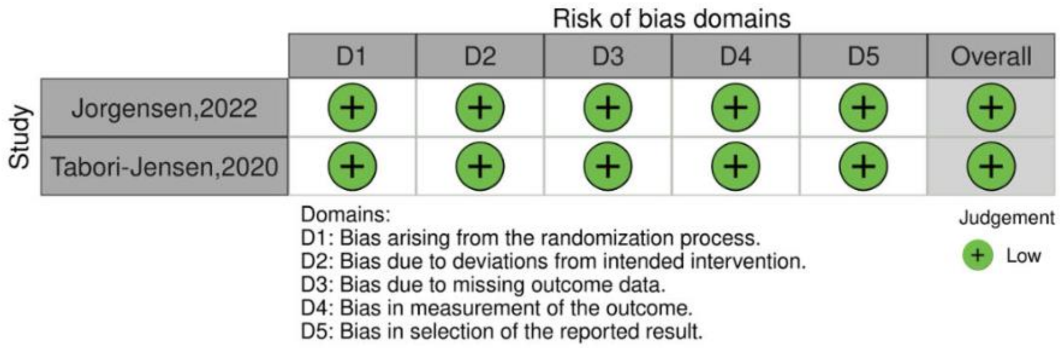

SUPPLEMENTARY FIGURE 1b. Cochrane Risk-of-Bias applied in randomized studies of interventions (**ROB 2**)

SUPPLEMENTARY FIGURE 2. Grade Assessment showing moderate certainty for Functional Score and high for Pain at rest, Pain during activity, Cup migration and Wear rate.

Dual Mobility Cemented compared to cementless cups in Total Hip Arthroplasty

Patient or population: Total Hip Arthroplasty
Setting: primary hip arthroplasty
Intervention: Dual Mobility Cemented
Comparison: cementless cups

Outcomes	No of participants (studies) Follow-up	Certainty of the evidence (GRADE)	Relative effect (95% CI)	Anticipated absolute effects: Risk with cementless cups	Anticipated absolute effects: Risk difference with Dual Mobility Cemented
Pain during activity	(2 RCTs)	High ⊕⊕⊕⊕	-	-	SMD 0.36 SD higher (0.03 lower to 0.74 higher)
Pain at rest	(2 RCTs)	High ⊕⊕⊕⊕	-	-	SMD 0.17 SD higher (0.21 lower to 0.56 higher)
Functional Score	(2 RCTs + 1 Observational)	Moderate ⊕⊕⊕	-	The median functional Score was 1.72	MD 1.72 higher (0.51 lower to 3.95 higher)
Cup migration	(2 RCTs)	Moderate ⊕⊕⊕	-	The mean cup migration was -0.45 SMD	0.45 SMD lower (0.96 lower to 0.05 higher)
Wear Rate	(2 RCTs)	Moderate ⊕⊕⊕	-	The median wear Rate was -0.13	MD 0.13 lower (0.4 lower to 0.13 higher)

*The risk in the intervention group (and its 95% confidence interval) is based on the assumed risk in the comparison group and the relative effect of the intervention (and its 95% CI).
CI: confidence interval; MD: mean difference; SMD: standardised mean difference
GRADE Working Group grades of evidence
High certainty: we are very confident that the true effect lies close to that of the estimate of the effect.
Moderate certainty: we are moderately confident in the effect estimate: the true effect is likely to be close to the estimate of the effect, but there is a possibility that it is substantially different.
Low certainty: our confidence in the effect estimate is limited: the true effect may be substantially different from the estimate of the effect.
Very low certainty: we have very little confidence in the effect estimate: the true effect is likely to be substantially different from the estimate of effect.
Explanations:
a. wide interval control

SUPPLEMENTARY TABLE 1. Search strategies for each database.

**Database**	**Search strategy**
Pubmed	("dual mobility" OR "dual-mobility" OR mobility[tiab]) AND (cementless[tiab] OR cemented[tiab] OR uncemented[tiab]) AND (cups[tiab] OR acetabular*[tiab] OR "acetabular system")
Embase	("dual mobility" OR "dual-mobility" OR mobility[tiab]) AND (cementless[tiab] OR cemented[tiab] OR uncemented[tiab]) AND (cups[tiab] OR acetabular*[tiab] OR "acetabular system")
Cochrane	("dual mobility" OR "dual-mobility" OR mobility[tiab]) AND (cementless[tiab] OR cemented[tiab] OR uncemented[tiab]) AND (cups[tiab] OR acetabular*[tiab] OR "acetabular system")
